# Heightened noise sensitivity as a predictor of psychological prognosis in older adults with traumatic brain injury

**DOI:** 10.3389/fpsyt.2025.1629337

**Published:** 2025-08-29

**Authors:** Huili Liu, Jingzhou Li

**Affiliations:** Department of Critical Care Medicine, The First People’s Hospital of Shangqiu, Shangqiu, Henan, China

**Keywords:** anxiety, depression, noise hypersensitivity, older adults, traumatic brain injury

## Abstract

**Background:**

Older adults are prone to falls, which may lead to traumatic brain injury, and the resulting psychological problems are also quite prominent. The purpose of this study is to explore the predictive role of recent noise hypersensitivity after mild traumatic brain injury (mTBI) in the risk of psychological problems such as depression and anxiety in the medium and long term.

**Methods:**

A total of 1,003 older adults (60 years old or older) with mTBI were included. At the short-term (2 weeks), medium-term (3 months), and long-term (12 months) after injury, the Rivermead Post-Concussion Symptoms Questionnaire, Hamilton Depression Scale, and Hamilton Anxiety Scale were used to assess patients’ noise hypersensitivity, depression, and anxiety symptoms, respectively. According to the short-term noise hypersensitivity, all patients were divided into the noise-sensitive group (n=441) and the non-sensitive group (n=592), and multivariate logistic regression was used to evaluate the association of noise hypersensitivity with depression and anxiety.

**Results:**

Short-term noise hypersensitivity after injury was correlated with medium-term and long-term depressive symptoms after injury (OR=1.063, 95%CI=1.027~1.100, P=0.001; OR=1.039, 95%CI=1.016~1.063, P=0.001), but not with short-term depressive symptoms after injury (OR=1.027, 95%CI=0.980~1.076, P=0.259). Short-term noise hypersensitivity was correlated with medium-term and long-term anxiety symptoms (OR=1.048, 95%CI=1.015~1.083, P=0.004; OR=1.042, 95%CI=1.013~1.073, P=0.004), but not with short-term anxiety symptoms (OR=1.034, 95%CI=0.991~1.078, P=0.128).

**Conclusion:**

Early-stage noise hypersensitivity after mTBI in older adults links to higher risk of mid- and late-stage adverse psychological prognosis (depression or anxiety). Whether noise hypersensitivity can predict post-injury psychological status in this population requires further verification.

## 1 Introduction

Currently, traumatic brain injury (TBI) has become the third major cause of the global disease burden, among which mild traumatic brain injury (mTBI) cases account for the majority (1). In the United States and China, there are hundreds of thousands or even millions of newly diagnosed TBI patients every year (2, 3). It should be emphasized that due to the increased risk of falls caused by declining physical functions, older adults are among the high-risk groups for TBI (4).

According to statistics, approximately 30% to 50% of mTBI patients will experience depressive symptoms, and about 20% to 40% of the patients will have anxiety-related symptoms after the injury (5–7). Currently, while there is no evidence indicating that older adults with TBI are at a higher risk of developing psychological disorders than middle-aged and young patients, the rehabilitation process for older adults is inherently slower and more challenging (8). If superimposed with psychological issues, it will further reduce their treatment initiative, shrink their social circle, and exacerbate the deterioration of cognitive and other functions, forming a vicious cycle of mutual influence between psychological and physical health. So, compared with middle-aged and young patients, psychological disorders in older adult TBI patients have a more critical impact on rehabilitation treatment and pose greater harms (8). In light of this, it is necessary to conduct in-depth research on the influencing factors of post-TBI psychological disorders in this population, so as to provide a basis for early prevention and intervention.

Noise hypersensitivity is also a common symptom following mTBI, with an incidence rate ranging from 20% to 80% (9–12). There are two hypotheses regarding the pathogenesis of this symptom (13, 14). The “information-processing hypothesis” suggests that TBI impairs the patient’s sound-processing system, rendering it unable to filter out irrelevant sounds and thereby leading to “auditory information overload.” The “negative affect hypothesis” posits that patients with noise hypersensitivity tend to be more critical and complain more readily. As we all know, somatic symptoms such as headache and dizziness primarily emerge during the acute phase of mTBI and are associated with physical trauma. In contrast, non-somatic symptoms such as sleep disturbances and cognitive impairments are influenced by multiple factors, including stress, age, underlying diseases, and environmental conditions, and thus have relatively lower specificity. Conversely, noise hypersensitivity may persist independently over the long term. It is most likely rooted in the brain’s excessive processing of auditory signals, belonging to a form of central sensitivity abnormality that directly involves the brain’s information processing system or arises from abnormalities in central emotional regulation. Thus, compared with other symptoms, noise hypersensitivity may have a specific association with psychological states, which could give it potential value in assessing the psychological prognosis of mTBI.

Recently, some studies have reported that noise hypersensitivity after TBI has prognostic significance and can predict the long-term presence of post-concussion symptoms and a decrease in the quality of life (15–17). Two other studies have explored the correlation between noise hypersensitivity and the psychological prognosis of TBI, yielding relatively conflicting results. One cross-sectional study indicated that noise hypersensitivity in TBI patients is significantly associated with anxiety symptoms (18), whereas another cohort study suggested that early noise hypersensitivity after TBI has no independent predictive value for long-term psychological outcomes (19). It is noteworthy that the sample sizes of these two studies are only 151 and 186 cases, respectively. Compared with the large population of TBI patients in the current study, their statistical power may be insufficient. These limitations create a critical knowledge gap—the true relationship between noise hypersensitivity and long-term psychological prognosis in TBI patients remains unclear—and thus underscore the necessity of conducting well-designed longitudinal follow-up studies with larger sample sizes to explore this topic in depth.

Therefore, this study aimed to include more than 1,000 older adults with mTBI to explore the correlation between noise hypersensitivity and the medium- and long-term psychological prognosis after the injury. The reasons for focusing on mTBI patients rather than those with moderate to severe TBI are as follows: (1) Mild cases constitute the majority of TBI patients; (2) To reduce the interference of severe physical injury on the assessment of psychological problems. The findings of this study may provide potential reference for identifying older adults with TBI at risk of adverse psychological outcomes, facilitating more precise early screening and targeted monitoring of high-risk groups in clinical practice. Additionally, they may offer insights for developing psychological intervention strategies for such older individuals, aiding in improving their psychological prognosis and optimizing rehabilitation outcomes through more timely and effective measures.

## 2 Materials and methods

### 2.1 Ethics requirements

This study was approved by a local authoritative medical ethics committee (ID: HS2023027). All subjects or their immediate family members agreed to participate in this study and signed the informed consent forms.

### 2.2 Subjects

From January 1, 2019, to March 31, 2023, older adult patients with mTBI were prospectively recruited from the Department of Critical Care Medicine of a major local medical institution. During this period, all patients who attended the department and met the inclusion criteria were enrolled in this study. It should be emphasized that the inclusion criteria were established prior to the initiation of recruitment and remained unchanged throughout the recruitment period. The specific items are as follows: (1) The age is greater than or equal to 60 years old. (2) Meet the diagnosis of mTBI, that is, there is a clear history of head trauma, no loss of consciousness or the duration of it does not exceed 30 minutes, no alteration of consciousness or the duration of it is less than 24 hours, no post-traumatic amnesia or the duration of it is less than 24 hours, Glasgow Coma Scale (GCS) score is 13 to 15, and cranial imaging examinations show no abnormality or only minor abnormalities (20). (3) Have had no psychiatric disorders before mTBI and have not taken any psychotropic drugs. (4) Agree to participate in the survey in the form of interviews, consent to provide medical records, and be able to complete 12-month follow-ups. (5) Have no other serious diseases (such as malignant tumors, severe infections, etc.), chronic diseases (such as hypertension and coronary heart disease) are stable, and there is no serious health risk in participating in this study. Subjects who did not meet the above criteria were directly excluded from this study.

Thus, a total of 1,079 patients met the above criteria and were enrolled at baseline. However, 46 patients were excluded from the study due to death during follow-up, refusal to participate in the survey, loss to follow-up, or failure to complete symptom assessments at the specified time points. Ultimately, 1,033 older adult patients with mTBI completed the entire study process.

### 2.3 Baseline data collection

Baseline data (including the basic characteristics of each patient and the characteristics of mTBI) were obtained from medical records during the patient’s hospitalization. Unclear or incomplete information was clarified by asking the attending doctor or family members. The detailed items of this information are listed in **Tables 1**, **2**.

**Table 1:** Baseline characteristics of patients in the noise-sensitive group and the non-sensitive group.

Variable	Noise-sensitive (n = 441) ^2^	Non-sensitive (n = 592)	t/χ² value	P value ^3^
Total (n)	441 (100.0)	592 (100.0)	-	-
Gender (n)				
Male	242 (54.9)	387 (65.4)	11.692	0.001
Female	199 (45.1)	205 (34.6)		
Age (years)	65.4 ± 3.1	65.1 ± 3.0	1.932	0.054
Education (n)				
≥12 years	217 (49.2)	245 (41.4)	6.254	0.012
<12 years	224 (50.8)	347 (58.6)		
Ethnic group (n)				
Han Chinese	410 (93.0)	538 (90.9)	1.465	0.226
Others	31 (7.0)	54 (9.1)		
Marital status (n)				
Married	243 (55.1)	356 (60.1)	2.628	0.105
Other	198 (44.9)	236 (39.9)		
Residence (n)				
Urban	331 (75.1)	426 (72.0)	1.238	0.266
Rural	110 (24.9)	166 (28.0)		
Chronic diseases (n)				
Diabetes	77 (17.5)	85 (14.4)	1.839	0.175
Hypertension	241 (54.6)	278 (47.0)	5.977	0.014
CHD^1^	106 (24.0)	114 (19.3)	3.444	0.063
CVD	48 (10.9)	47 (7.9)	2.625	0.105
Thyroid dysfunction	71 (16.1)	76 (12.8)	2.203	0.138

^1^CHD, Coronary Heart Disease; CVD, Cerebrovascular Disease. ^2^Continuous variables are expressed as mean ± standard deviation, and categorical variables are expressed as frequency (constituent ratio). ^3^A P-value less than 0.05 indicates that the difference is statistically significant.

**Table 2:** Characteristics of mild traumatic brain injury in the noise-sensitive group and the non-sensitive group.

Variable	Noise-sensitive (n = 441) ^2^	Non-sensitive (n = 592)	t/χ² value	P value ^3^
Total (n)	441 (100.0)	592 (100.0)	-	-
GCS score (n) ^1^				
13 scores	110 (24.9)	126 (21.3)	1.920	0.166
14 scores	134 (30.4)	161 (27.2)	1.260	0.262
15 scores	197 (44.7)	305 (51.5)	4.746	0.029
Loss of consciousness				
No. of patients (n)	206 (46.7)	235 (39.7)	5.085	0.024
Duration (minute)	9.2 ± 5.3	8.1 ± 4.8	2.240	0.026
Posttraumatic amnesia				
No. of patients (n)	192 (43.5)	217 (36.7)	5.005	0.025
Duration (hour)	10.7 ± 5.6	9.4 ± 5.0	2.386	0.017
Abnormal brain CT (n)	57 (12.9)	60 (10.1)	1.959	0.162
Causes of trauma (n)				
Fall	220 (49.9)	290 (49.0)	0.082	0.775
Automobile	119 (27.0)	183 (30.9)	1.885	0.170
Other accidents	102 (23.1)	119 (20.1)	1.378	0.240
Previous TBI (n)				
Absent	374 (84.8)	527 (89.0)	4.025	0.045
Present	67 (15.2)	65 (11.0)		

^1^GCS, Glasgow Coma Scale; CT, Computed Tomography; mTBI, Mild traumatic brain injury. ^2^Continuous variables are expressed as mean ± standard deviation, and categorical variables are expressed as frequency (constituent ratio). ^3^A P-value less than 0.05 indicates that the difference is statistically significant.

### 2.4 Symptom scores

At 2 weeks post-injury, acute symptoms such as headache and dizziness have gradually stabilized, making noise hypersensitivity more prominent or more easily noticeable to patients, while psychological issues at this stage are primarily associated with traumatic stress. By 3 months post-injury, acute symptoms have largely subsided, and persistent psychological symptoms (e.g., anxiety, depression) begin to emerge. At 12 months post-injury, these persistent psychological symptoms have stabilized, facilitating the assessment of long-term prognosis. Thus, these time points are defined as the short-term, medium-term, and long-term post-injury periods, respectively.

Based on this, noise hypersensitivity symptoms in each patient were evaluated using the Rivermead Post-Concussion Symptoms Questionnaire (RPCSQ) at 2 weeks, 3 months, and 12 months post-injury, respectively (21). This scale is specifically designed for assessing post-concussion symptoms and has been widely recognized (22). To meet the needs of this study, necessary supplements were made to the section related to hyperacusis within the scale. Specifically:

- A score of 0 indicates the absence of hyperacusis, with no perception of harshness from any normal sounds.
- A score of 1 indicates the presence of hyperacusis, occurring less than once per week, where only a few normal sounds are perceived as slightly harsh but remain tolerable, without interfering with normal auditory activities.
- A score of 2 indicates hyperacusis occurring 1 to 2 times per week, where some normal sounds are perceived as relatively harsh, requiring slight distancing from the sound source but without impairing normal auditory communication.
- A score of 3 indicates hyperacusis occurring 3 to 5 times per week, where many normal sounds are perceived as harsh, necessitating avoidance of certain noisy environments and exerting a moderate impact on normal auditory communication.
- A score of 4 indicates hyperacusis occurring almost daily, where most normal sounds are intolerable, severely impairing auditory communication and potentially requiring protective measures such as earplugs.

Examples of normal sounds include but are not limited to the inflation sound of a blood pressure cuff, the operational sound of nurses replacing infusion bags, regular alerts from ECG monitors, walking sounds, mild coughing sounds, and medium-speed running water from a tap.

Also at these three post-injury time points, the depressive and anxious symptoms of each patient were evaluated respectively using the 17-item version of the Hamilton Depression Scale (HAMD-17) and the 14-item version of the Hamilton Anxiety Scale (HAMA-14) (23, 24). The HAMD-17 contains 17 items, and the total score ranges from 0 to 52. Scores <7 indicate no depressive symptoms; 7 to 17, mild depression; 18 to 24, moderate depression; and ≥25, severe depression. The HAMA-14 contains 14 items, and the total score ranges from 0 to 56. Scores <7 indicate no anxiety symptoms; 7 to 13: possible anxiety; 14 to 20: definite anxiety; 21 to 28: obvious anxiety; >28: severe anxiety.

In addition, at 2 weeks after the injury, the RPCSQ was also used to evaluate other post-mTBI symptoms of each patient, including headache, dizziness, nausea, vomiting, balance disorder, vision problems, inattention, memory loss, and sleep disorders (**Table 3**). The scale for most symptoms is divided into five levels (i.e., 0, 1, 2, 3, and 4 points). The higher the score, the more severe the symptom. It should be specially noted that “vision problems” actually include blurred vision and light sensitivity, and “sleep disorders” include difficulty in falling asleep, excessive dreaming, and easy awakening. In this study, vision problems and sleep disorders were respectively analyzed as a whole. Their score ranges are 0 to 8 points and 0 to 12 points, respectively. Although these composite scores were not separately validated, their sub-items are derived from the validated scale with consistent scoring methods, and the results in **Table 3** confirm their effectiveness in reflecting inter-group differences. This approach aligns with symptom characteristics in older adults with mTBI and provides a reasonable simplification for analyses.

**Table 3:** Symptoms two weeks after mild traumatic brain injury in the noise-sensitive group and the non-sensitive group.

Variable (No. of cases)	Noise-sensitive (n = 441) ^1^	Non-sensitive (n = 592)	t/χ² value	P value ^2^
Headache	242 (54.9)	283 (47.8)	5.056	0.025
Dizziness	233 (52.8)	274 (46.3)	4.340	0.037
Nausea	154 (34.9)	186 (31.4)	1.403	0.236
Vomiting	86 (19.5)	101 (17.1)	1.015	0.314
Balance disorder	79 (17.9)	94 (15.9)	0.751	0.386
Vision problems	155 (35.1)	172 (29.1)	4.337	0.037
Inattention	265 (60.1)	319 (53.9)	3.961	0.047
Memory loss	183 (41.5)	210 (35.5)	3.890	0.049
Sleep disorders	177 (40.1)	195 (32.9)	5.681	0.017
Variable (Symptom scores)	Noise-sensitive (n = 441)	Non-sensitive (n = 592)	t/χ² value	P value
Headache	1.10 ± 1.13	0.90 ± 1.03	2.969	0.003
Dizziness	1.05 ± 1.12	0.89 ± 1.07	2.295	0.022
Nausea	0.67 ± 1.00	0.63 ± 1.00	0.643	0.521
Vomiting	0.38 ± 0.84	0.33 ± 0.78	1.061	0.289
Balance disorder	0.36 ± 0.82	0.33 ± 0.80	0.600	0.548
Vision problems	1.24 ± 1.89	0.88 ± 1.55	3.323	0.001
Inattention	1.20 ± 1.11	1.03 ± 1.09	2.531	0.012
Memory loss	0.82 ± 1.07	0.71 ± 1.05	1.529	0.127
Sleep disorders	2.05 ± 2.95	1.68 ± 2.76	2.069	0.039

^1^Continuous variables are expressed as mean ± standard deviation, and categorical variables are expressed as frequency (constituent ratio). ^2^A P-value less than 0.05 indicates that the difference is statistically significant.

These assessments were conducted by specialized investigators through in-hospital evaluations or home visits. Each assessment was performed jointly by two investigators, who independently recorded their results. Both sets of results were submitted to data analysts for verification to ensure data completeness and consistency in a timely manner. In cases of incomplete or inconsistent data, the data analysts would directly contact the patients or their family members for verification.

To further validate inter-rater reliability, the post-injury 2-week symptom assessment data of 200 randomly selected subjects were extracted, and kappa coefficients were calculated by data analysts. The results showed that the kappa values for noise hypersensitivity, anxiety, and depression scores were 0.81, 0.79, and 0.83, respectively (all P<0.001), indicating good agreement between the two investigators in their assessments.

It should be emphasized that the nature of this study precluded the implementation of effective double-blind or triple-blind procedures; however, blinding methods were employed to the extent possible. Briefly, there was no communication between the investigators conducting symptom assessments and those responsible for data analysis. This ensured that the assessing investigators were unlikely to have accurate knowledge of the participants’ grouping or other relevant information during symptom evaluations, thereby enabling more objective recording of assessment data and minimizing observer bias as much as possible.

### 2.5 Statistical analysis

All patients were divided into the noise-sensitive group (with scores ranging from 1 to 4) and the non-sensitive group (all with a score of 0) according to the noise-hypersensitivity scores two weeks after injury, including 441 and 592 older adult patients with mTBI respectively.

Continuous variables are expressed as means ± standard deviations. The difference in each variable between the two groups was evaluated by an independent-samples t-test, and the t-value and P-value were reported. Categorical variables are expressed as frequency and proportion. The difference in each variable between the two groups was evaluated by a chi-square test, and the χ²-value and P-value were reported. The linear relationship between the noise-hypersensitivity score and the psychological score was evaluated by Pearson’s linear correlation, and the r-value and P-value were reported.

The multivariate correlation between the noise-hypersensitivity score and the psychological score was evaluated by multivariate logistic regression, and the odds ratio (OR), 95% confidence interval (95%CI) and P-value were reported. In the multivariate model, the following potential confounding factors were adjusted for (as shown in **Tables 1**-**3**): (1) Patients’ baseline characteristics, including gender, age, education level, ethnic group, marital status, residence, and history of chronic diseases; (2) mTBI characteristics, including GCS score, loss of consciousness, post-traumatic amnesia, brain CT findings, causes of trauma, and history of previous TBI; (3) Other symptoms at 2 weeks post-injury, including headache, dizziness, nausea, vomiting, balance disorder, vision problems, inattention, memory loss, and sleep disorders. In all the above analyses, a P-value<0.05 indicates that the difference or correlation is statistically significant. In addition, multicollinearity among covariates was assessed using variance inflation factors (VIFs), and the goodness-of-fit of the logistic regression models was evaluated via the Hosmer-Lemeshow test. All analyses mentioned above were performed using SPSS 27.0.

It is important to note that Cox proportional hazards regression was not used in this study, primarily for the following reasons: All participants completed identical follow-up periods and were assessed at fixed time points—specifically, all underwent evaluations for noise hypersensitivity and psychological measurements at 2 weeks, 3 months, and 12 months post-injury, as pre-specified. The outcome variables were defined as “whether a specific event occurred at a given time point” rather than “the time elapsed from injury to event occurrence,” making the data categorical rather than time-to-event in nature. Any participants who failed to complete assessments at the designated time points were excluded from the study. Consequently, multivariate logistic regression was better suited for analyzing the association between exposure at a fixed time point (short-term noise sensitivity) and outcomes (medium- and long-term psychological symptoms).

## 3 Results

### 3.1 Baseline characteristics of patients

In **Table 1**, compared with the non-sensitive group, the proportions of females, those with education≥12 years, and those suffering from hypertension were higher in the noise-sensitive group (P=0.001, P=0.012, P=0.014). Potential explanations for these results are as follows: Females tend to have greater sensitivity to sensory stimuli; individuals with higher education may pay more attention to their own symptoms; and vascular lesions caused by hypertension may affect cerebral blood flow, thereby exacerbating the abnormal sensitivity of the auditory center. These findings provide a comprehensive and multi-dimensional perspective for understanding the clinical characteristics of noise hypersensitivity.

In addition, there were no statistically significant differences between the two groups in terms of the other characteristics (P>0.05).

### 3.2 Characteristics of mTBI

In **Table 2**, the proportion of a GCS score of 15 was lower in the noise-sensitive group than in the non-sensitive group (P=0.029). The proportions of having loss of consciousness and post-traumatic amnesia were higher in the noise-sensitive group than in the non-sensitive group (P=0.024, P=0.025), and the average durations of these two symptoms were longer in the noise-sensitive group (P=0.026, P=0.017). The proportion of having a previous TBI was also higher in the noise-sensitive group than in the non-sensitive group (P=0.045). In addition, there were no statistically significant differences between the two groups in terms of abnormal brain CT and causes of trauma (All P>0.05).

### 3.3 Symptoms two weeks after mTBI

In **Table 3**, a series of post-mTBI symptoms were evaluated two weeks after injury. The proportions of headache, dizziness, vision problems, inattention, memory loss and sleep disorders were all higher in the noise-sensitive group than in the non-sensitive group (P=0.025, P=0.037, P=0.037, P=0.047, P=0.049, P=0.017). Except for memory loss (P=0.127), the scores of the other five symptoms were also higher in the noise-sensitive group than in the non-sensitive group (P=0.003, P=0.022, P=0.001, P=0.012, P=0.039). In addition, there were no statistically significant differences between the two groups in terms of the proportions of patients and scores of nausea, vomiting and balance disorder (All P>0.05).

### 3.4 Noise hypersensitivity and psychological prognosis

In **Table 4**, the study assessed noise hypersensitivity and psychological prognosis at 2 weeks, 3 months and 12 months after injury respectively.

**Table 4:** Hypersensitivity to noise and psychological prognosis after mild traumatic brain injury in the noise-sensitive group and the non-sensitive group.

Variable (No. of cases)	Noise-sensitive (n = 441) ^2^	Non-sensitive (n = 592)	t/χ² value	P value ^3^
Sensitivity to noise (n)				
Two weeks after mTBI ^1^	441 (100.0)	0	-	-
Three months after mTBI	390 (88.4)	79 (13.3)	574.886	<0.001
Twelve months after mTBI	328 (74.4)	60 (10.1)	444.731	<0.001
Depression (n)				
Two weeks after mTBI	40 (9.1)	42 (7.1)	1.350	0.245
Three months after mTBI	104 (23.6)	102 (17.2)	6.389	0.011
Twelve months after mTBI	207 (46.9)	225 (38.0)	8.287	0.004
Anxiety (n)				
Two weeks after mTBI	75 (17.0)	72 (12.2)	4.860	0.027
Three months after mTBI	120 (27.2)	119 (20.1)	7.183	0.007
Twelve months after mTBI	160 (36.3)	162 (27.4)	9.365	0.002
Variable (Symptom scores)	Noise-sensitive (n = 441)	Non-sensitive (n = 592)	t/χ² value	P value
Sensitivity to noise				
Two weeks after mTBI	1.54 ± 0.82	0	-	-
Three months after mTBI	1.42 ± 0.93	0.19 ± 0.53	26.917	<0.001
Twelve months after mTBI	1.15 ± 0.95	0.13 ± 0.42	23.367	<0.001
Depression				
Two weeks after mTBI	3.68 ± 2.65	3.48 ± 2.64	1.149	0.251
Three months after mTBI	5.07 ± 4.20	4.24 ± 3.32	3.565	<0.001
Twelve months after mTBI	7.37 ± 6.05	6.19 ± 5.19	3.378	0.001
Anxiety				
Two weeks after mTBI	3.80 ± 2.94	3.52 ± 2.86	1.545	0.123
Three months after mTBI	5.32 ± 4.26	4.63 ± 3.42	2.885	0.004
Twelve months after mTBI	5.92 ± 4.86	5.12 ± 3.94	2.897	0.004

^1^mTBI, Mild traumatic brain injury. ^2^Continuous variables are expressed as mean ± standard deviation, and categorical variables are expressed as frequency (constituent ratio). ^3^A P-value less than 0.05 indicates that the difference is statistically significant.

In terms of noise hypersensitivity, at 3 and 12 months post-injury, both the incidence of noise hypersensitivity and the symptom score levels in the noise-sensitive group were significantly higher than those in the non-sensitive group (3 months post-injury: P<0.001, P<0.001; 12 months post-injury: P<0.001, P<0.001), suggesting that noise hypersensitivity is persistent in the sensitive population.

Regarding depression, at 2 weeks post-injury, there were no significant differences in the incidence of depression or the inter-group symptom score levels between the two groups (P=0.245, P=0.251), and the mean symptom scores in both groups were below the threshold for mild depression (7 points). At 3 and 12 months post-injury, the incidence of depression and the symptom score levels in the noise-sensitive group were significantly higher than those in the non-sensitive group (3 months post-injury: P=0.011, P<0.001; 12 months post-injury: P=0.004, P=0.001). The mean symptom scores in both groups showed an increasing trend across the three time points, with the increase appearing more pronounced in the noise-sensitive group. At 12 months post-injury, the mean symptom score in this group exceeded the threshold for mild depression (7 points).

For anxiety, at 2 weeks post-injury, there was no significant difference in the symptom score levels between the two groups (P=0.123), but the incidence of anxiety in the noise-sensitive group was higher than that in the non-sensitive group (P=0.027), and the mean symptom scores in both groups were below the threshold for possible anxiety (7 points). At 3 and 12 months post-injury, the incidence of anxiety and the symptom score levels in the noise-sensitive group were significantly higher than those in the non-sensitive group (3 months post-injury: P=0.007, P=0.004; 12 months post-injury: P=0.002, P=0.004). The mean symptom scores in both groups also showed an increasing trend across the three time points, with the highest mean score recorded in the noise-sensitive group at 12 months post-injury, which was 5.92 points, approaching the threshold for possible anxiety (7 points).

It can be seen that compared with the non-sensitive group, the two psychological symptoms in the noise-sensitive group are more significantly aggravated, as evidenced by a higher number of people suffering from depression or potential anxiety and higher mean symptom scores. These univariate analysis findings tentatively hint that individuals in the noise-sensitive group could potentially benefit from additional psychological assessments or supportive measures, though further investigation would be needed to confirm such needs.

### 3.5 Linear correlation analysis

**Figure 1:**
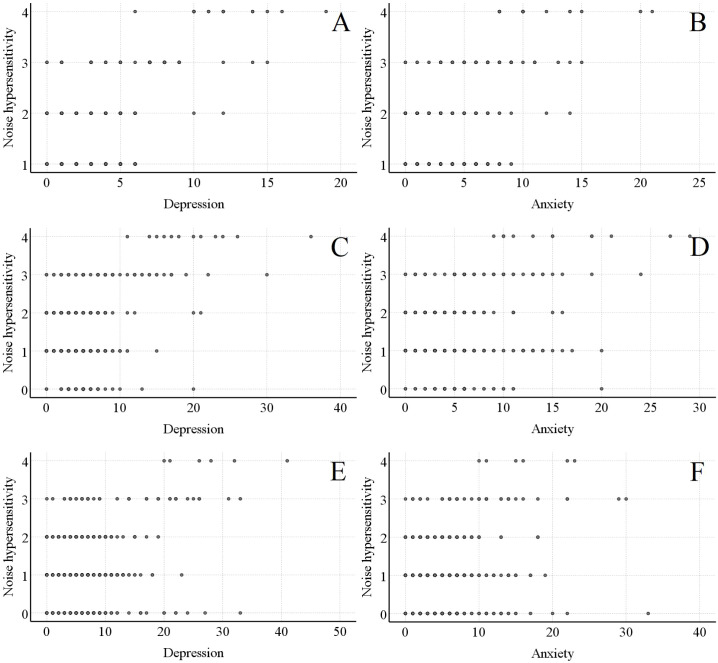
Linear relationship between noise - sensitive scores and psychological prognosis scores in the noise - sensitive group. **(A, B)** respectively show the linear relationships between noise - sensitive scores and psychological scores 2 weeks after mild traumatic brain injury. **(C, D)** respectively show the linear relationships between noise - sensitive scores and psychological scores 3 months after mild traumatic brain injury. **(E, F)** respectively show the linear relationships between noise - sensitive scores and psychological scores 12 months after mild traumatic brain injury.

In **Figures 1A, C, E**, at 2 weeks, 3 months, and 12 months post-injury, there was a significant positive linear relationship between noise hypersensitivity scores and depression scores (r=0.510, P<0.001; r=0.399, P<0.001; r=0.287, P<0.001). In **Figures 1B, D, F**, noise hypersensitivity scores also exhibited a significant positive linear relationship with anxiety scores at these three time points (r=0.424, P<0.001; r=0.298, P<0.001; r=0.190, P<0.001). These results could be interpreted as loosely pointing to a potential dose-response pattern between noise hypersensitivity and psychological status, though such an association remains speculative. It should be noted, however, that these tentative observations stem from data at only three time points, and their robustness would need to be explored through more extensive research.

Notably, the aforementioned linear analyses were conducted only in the noise-sensitive group, as noise hypersensitivity scores in the non-sensitive group were either 0 or predominantly 0 at all three time points.

### 3.6 Multivariate analysis

In **Table 5**, the multivariate model showed that noise hypersensitivity two weeks after injury was significantly correlated with depressive symptoms three and twelve months after injury (OR=1.063, 95%CI=1.027~1.100, P=0.001; OR=1.039, 95%CI=1.016~1.063, P=0.001), but not with depressive symptoms two weeks after injury (OR=1.027, 95%CI=0.980~1.076, P=0.259). Similarly, noise hypersensitivity two weeks after injury was significantly correlated with anxiety symptoms three and twelve months after injury (OR=1.048, 95%CI=1.015~1.083, P=0.004; OR=1.042, 95%CI=1.013~1.073, P=0.004), but not with anxiety symptoms two weeks after injury (OR=1.034, 95%CI=0.991~1.078, P=0.128).

**Table 5:** Multivariate Correlation between hypersensitivity to noise and psychological prognosis after mild traumatic brain injury in the noise-sensitive group and the non-sensitive group.

Variable ^1^	Univariate model OR (95%CI)	P value ^2^
Noise hypersensitivity two weeks after mTBI		
Depression two weeks after mTBI	1.028 (0.981 ~ 1.077)	0.251
Depression three months after mTBI	1.062 (1.027 ~ 1.099)	0.001
Depression twelve months after mTBI	1.039 (1.016 ~ 1.063)	0.001
Anxiety two weeks after mTBI	1.034 (0.991 ~ 1.079)	0.124
Anxiety three months after mTBI	1.049 (1.015 ~ 1.083)	0.004
Anxiety twelve months after mTBI	1.042 (1.013 ~ 1.073)	0.004
Variable	Multivariate model ^2^ OR (95%CI)	P value ^3^
Noise hypersensitivity two weeks after mTBI		
Depression two weeks after mTBI	1.027 (0.980 ~ 1.076)	0.259
Depression three months after mTBI	1.063 (1.027 ~ 1.100)	0.001
Depression twelve months after mTBI	1.039 (1.016 ~ 1.063)	0.001
Anxiety two weeks after mTBI	1.034 (0.991 ~ 1.078)	0.128
Anxiety three months after mTBI	1.048 (1.015 ~ 1.083)	0.004
Anxiety twelve months after mTBI	1.042 (1.013 ~ 1.073)	0.004

^1^mTBI, Mild traumatic brain injury; OR, Odds Ratio; 95%CI, 95% confidence interval. ^2^Multivariate model adjusted for the baseline characteristics of the subjects, the characteristics of mild traumatic brain injury, and other symptoms two weeks after the injury. ^3^A P-value less than 0.05 indicates that the multivariate correlation is statistically significant.

For multicollinearity assessment, VIFs were calculated for all covariates included in the multivariate logistic regression models. The VIF values ranged from 1.03 to 1.89 (all<2.0), indicating no significant multicollinearity among the covariates. Regarding model goodness-of-fit, the Hosmer-Lemeshow test was performed for each logistic regression model. The results showed non-significant chi-square statistics (all P>0.05), suggesting that the observed data were consistent with the predictions of the models, indicating acceptable goodness-of-fit.

These results indicate that short-term noise hypersensitivity after injury is at least statistically associated with mid- to long-term psychological disorders.

## 4 Discussion

This study demonstrates a statistically significant association between noise hypersensitivity at 2 weeks post-injury and a 4% to 6% increased risk of adverse mid- to long-term psychological outcomes. Despite the relatively clear temporal sequence between the two, this study employs an observational design. Therefore, the validity of noise hypersensitivity as a clinical predictive or screening indicator for psychological prognosis requires further verification through multi-level evidence, including clinical interventional studies, and basic experiments. Nevertheless, this study provides a crucial basis for future research in this area.

The multivariate model in this study adjusted for potential confounding factors as comprehensively as possible, including patients’ baseline characteristics (e.g., gender, chronic diseases), mTBI features (e.g., GCS score, duration of loss of consciousness), and other symptoms at 2 weeks post-injury (e.g., headache, sleep disorders). Notably, comparisons before and after adjustment revealed that the magnitude of change in the association strength (OR) between noise hypersensitivity and mid-to-long-term psychological prognosis was less than 5%, with no substantial impact on statistical significance. This indicates that the included covariates had only a minor effect on the overall results and did not significantly alter the core association between the two. We acknowledge that residual confounding may still exist—particularly given that noise hypersensitivity and other post-concussive symptoms may share a common etiology. However, collinearity tests showed no significant correlations among covariates (VIF<2.0), suggesting these variables are relatively independent in the model, which reduces the risk of residual confounding caused by overlapping associations between variables to some extent. Thus, these factors enhance the reliability of the results.

In the multivariate analysis, this study used noise hypersensitivity (as a binary variable) rather than noise hypersensitivity scores as the analytical variable, based primarily on the following considerations: On one hand, mild cases predominate in the noise-sensitive group of this study. For instance, at 2 weeks post-injury, 379 participants (85.9%) had noise hypersensitivity scores of 1 to 2, while only 62 participants (14.1%) scored 3 to 4. This highly imbalanced data distribution makes it challenging to analyze differences in symptom severity or explore dose-response relationships. On the other hand, the noise hypersensitivity scores range from 0 to 4, with scores of 1 to 4 all indicating the presence of noise hypersensitivity. However, current intervention strategies remain underdeveloped and cannot provide targeted measures for patients with different scores. As a result, in clinical practice, all patients with noise hypersensitivity receive conventional interventions such as environmental noise reduction and psychological counseling. Thus, dichotomizing the scores would not significantly compromise clinical decision-making. This study acknowledges that dichotomizing scores does result in some loss of information. Nevertheless, considering the data characteristics of this study and real-world clinical practice, this approach represents a balance between practicality and feasibility.

According to the results, early noise hypersensitivity is only associated with a 4% to 6% increase in the risk of adverse psychological outcomes. This finding requires comprehensive interpretation in the context of the pathogenesis of psychological disorders. Briefly, diseases such as depression and anxiety have complex etiologies involving numerous risk factors, and the independent role of any single factor is unlikely to be significant. Therefore, this magnitude of risk increase is clinically meaningful. As noted in the introduction, the baseline risk of psychological disorders in TBI patients is substantial: 30% to 50% may develop depressive symptoms, and 20% to 40% may experience anxiety symptoms. Against this backdrop, a 4% to 6% increase in risk further amplifies the likelihood of adverse outcomes. Currently, research on the association between noise hypersensitivity and psychological prognosis remains emerging, with no comparable predictors of larger effect sizes identified. Even if other influencing factors are discovered in the future, the association revealed in this study will still provide unique reference value for the early identification of high-risk populations.

From another perspective, although the observed association between short-term noise hypersensitivity and mid-to-long-term depressive and anxiety symptoms has a relatively small effect size, it offers a new lens for understanding the psychological prognosis of older adults with mTBI. In the context of patients’ recovery trajectories, persistent noise hypersensitivity may be linked to the protraction of psychological symptoms; given the unique rehabilitation characteristics of older individuals, this association could influence their overall recovery progress. In clinical practice, early post-injury monitoring of noise hypersensitivity is a promising area for assessment. For patients with such symptoms, trialing interventions like environmental modifications and psychological support may provide new approaches to mitigating mid-to-long-term psychological symptoms and optimizing rehabilitation strategies. These observations aim to provide preliminary insights for managing the psychological prognosis of older adults with mTBI, with their full clinical utility warranting further investigation.

In 2023, Marzolla et al. reported findings inconsistent with those of the present study (19). It is important to note that this study has several methodological strengths: a larger sample size (1,033 participants), strictly fixed follow-up time points (2 weeks, 3 months, and 12 months post-injury), and intentional exclusion of participants with a history of psychiatric disorders during recruitment. In contrast, the 2023 study included only 186 TBI patients, allowed a 4-week window for follow-up assessments (potentially introducing timing bias), and did not explicitly exclude individuals with pre-existing mental health conditions (which may confound post-injury psychological symptoms with prior psychiatric illness). These factors collectively afford this study certain advantages in design and methodology. Additionally, differences in psychological assessment scales and the age ranges of the study populations may partially explain the divergent results. Future research could further verify the causes of these discrepancies by standardizing assessment tools and follow-up procedures.

Despite efforts to refine the methodology, G*Power calculations revealed that the statistical power of this study remains far below the widely accepted standard (80%). We argue that low statistical power is not a failure of study design but an inevitable consequence of exploring “small-effect” associations. In this study, the “small effect” refers to the statistically significant but weak correlation between short-term noise hypersensitivity and mid-to-long-term psychological outcomes, as indicated by OR values very close to 1. As previously noted, psychological disorders are multifactorial, and noise hypersensitivity alone is likely to exert only a “small effect”—an objective reality. Furthermore, in multivariate analyses, such associations may be further diluted by confounding factors like age and underlying diseases, a challenge difficult to fully avoid in statistical analysis. However, the 1,033-participant sample size of this study represents a critical breakthrough, with undeniable advantages. Nevertheless, power remains limited due to the inherently small effect size of the association under investigation. In contrast, previous studies in this field with sample sizes of only 100 to 200 likely faced more severe power issues, potentially leading to complete omission of weak associations and reporting of false-negative results. This study is the first to stably capture a significant trend (P < 0.05) in the correlation between noise hypersensitivity and psychological outcomes in the mTBI population, marking a transition from “undetectable” to “first discovery”—a step forward in itself. This low statistical power underscores the challenges in this research area and points to future directions: validation with larger samples or exploration of effect modifiers through stratified analyses. This study provides a reference benchmark and feasible path for subsequent investigations.

Regarding the selection of noise hypersensitivity assessment tools, dedicated scales often include content related to social and occupational contexts, which are not particularly applicable to TBI patients during hospitalization or rehabilitation. Therefore, this study adapted the noise hypersensitivity item from the RPCSQ, covering three dimensions: frequency of noise hypersensitivity, breadth of triggering sounds, and impact on auditory communication. Additionally, sound examples relevant to TBI patients were added to meet the assessment needs of this population. To reduce subjective bias in assessments, two investigators participated independently and recorded results separately; subsequent kappa coefficient analysis confirmed sufficient agreement between their evaluations. These measures enhanced the reliability of the noise hypersensitivity assessment strategy. Of course, this study acknowledges that, compared to specialized noise hypersensitivity tools, this single item has limitations. However, the RPCSQ item reliably distinguishes between noise-sensitive and non-sensitive TBI patients, which is sufficient to support the conduct of this study. Nevertheless, future research should develop dedicated hyperacusis scales or feasible assessment methods for TBI patients.

The HAMD-17 and HAMA-14 also have limitations in assessing psychological status among older adults with mTBI. Briefly, their somatic items (e.g., fatigue, muscle tension, sleep disturbance) may overlap with age-related changes, chronic diseases, or TBI-specific neurological symptoms, complicating the accurate differentiation of true emotional/anxiety symptoms from somatic sequelae. However, as noted earlier, the multivariate models adjusted for numerous post-TBI somatic symptoms (e.g., headache, sleep disorders) and chronic disease history as confounding factors, and these adjustments did not affect the key findings—mitigating, to some extent, the interference from this limitation.

As mentioned, no studies have yet explored the mechanisms by which noise hypersensitivity promotes or predicts psychological disorders after mTBI. However, potential mechanisms can be inferred based on knowledge from related medical fields: (1) mTBI may cause mild damage to brain regions involved in psychological regulation, such as the frontal lobe, temporal lobe, or hippocampus (25, 26); noise stimulation may further impact these injured regions, triggering depression and anxiety. (2) The serotonin system, dopamine system, gamma-aminobutyric acid system, and hypothalamic-pituitary-adrenal axis are all involved in psychological regulation (27–30); these systems may be impaired in mTBI patients, and noise stimulation could exacerbate their dysfunction, increasing the risk of depression and anxiety. (3) Post-mTBI noise hypersensitivity may hinder social and family reintegration, work, and study, leading to self-doubt—factors that may also contribute to depression and anxiety. These inferences require further exploration.

In addition to the issues elaborated above, this study has the following limitations: (1) During follow-up, noise hypersensitivity was assessed at only three time points, leaving the detailed trajectory of this symptom and its prognostic significance for psychological outcomes unexplored. This is primarily because participants could not tolerate excessively frequent psychological assessments. Future research should develop more convenient and patient-friendly psychological monitoring methods to address this gap. (2) The study population consists exclusively of Chinese participants, significantly limiting the generalizability of the findings. It is crucial to emphasize that cultural contexts may profoundly affect result extrapolation. For example, tolerance thresholds for environmental noise and subjective interpretations of noise interference may vary across cultures; perceptions and expressions of psychological symptoms may differ between populations, and stigma around mental health may introduce reporting bias. Additionally, disparities in healthcare access across countries may alter the strength of the association between noise hypersensitivity and psychological prognosis. These factors suggest that the core conclusions may be population-specific, and future studies should include more diverse ethnic and cultural groups for validation.

In conclusion, this study confirms that early-onset noise hypersensitivity after mTBI in older adults may be associated with an increased risk of adverse mid-to-long-term psychological outcomes. Based on this finding, clinical practice can proactively optimize management strategies. For instance, noise hypersensitivity screening could be integrated into routine assessments of older mTBI patients at 2 weeks post-injury. Screen-positive individuals should be classified as high-risk for psychological disorders, with dynamic monitoring of depressive and anxiety symptoms within 12 months post-injury. Additionally, targeted interventions—including environmental noise control and personalized counseling—should be implemented early. These measures are expected to provide more precise rehabilitation care, effectively improving psychological prognosis and quality of life in older mTBI patients. This study offers an initial basis for refining such clinical pathways, and subsequent research should further validate their effectiveness to promote the development of standardized intervention protocols.

## Data Availability

The raw data supporting the conclusions of this article will be made available by the authors, without undue reservation.
